# Subjective costs and benefits of online self-help group participation: findings from qualitative research with family caregivers for individuals of Turkish descent living with dementia in Germany

**DOI:** 10.3389/frdem.2026.1763257

**Published:** 2026-05-21

**Authors:** Rona Bird, Leman Bilgic, Sharon Häußler, Zeynep Gırgırlar, Sümeyra Öztürk, Mualla Basyigit, Ela Rana Örs, Samuel Friedrich Hege, Sezen Çakmak, Anja Rutenkröger, Christina Kuhn, Kübra Annac, Patrick Brzoska, Yüce Yilmaz-Aslan, Hürrem Tezcan-Güntekin

**Affiliations:** 1 Faculty of Health and Education, Alice Salomon University of Applied Sciences, Berlin, Germany; 2 Demenz Support Stuttgart gGmbH Zentrum für Informationstransfer, Marco-Polo-Zentrum Zeppelinstr, Ostfildern, Germany; 3 Berlin School of Public Health, Charité Universitätsmedizin Berlin, Berlin, Germany; 4 Faculty of Humanities and Educational Sciences, Department of Educational Science - Teching and Learning in the Migration Society, Institute of Education, Technical University Berlin, Berlin, Germany; 5 Faculty of Health, School of Medicine, Health Services Research Unit, Witten/Herdecke University, Witten, Germany

**Keywords:** critical reflexivity, dementia, digital health, family caregiver, intersectionality, intervention, migration, racialized communities

## Abstract

**Introduction:**

Caring for a family member with dementia is associated with stressors across psychological, physical, social and economic domains. Caregivers from racialized communities face additional burdens of racism and exclusion, leading to barriers in utilizing services and support-programs. Health-promotion interventions for family caregivers can ease caregiver burden by providing support, decreasing loneliness and increasing self-efficacy. Addressing structural exclusion and intersectional discrimination is essential to ensure access for caregivers from racialized communities.

**Methods:**

This article presents findings from the qualitative evaluation of an online self-help program for family caregivers for individuals of Turkish descent living with dementia in Germany. Eight program participants were interviewed at two different time points. Additionally, eight interviews were conducted with individuals who declined to participate in the program for a qualitative study examining reasons for non-participation. All data were analyzed using structuring qualitative content analysis.

**Results:**

The evaluation and non-participation studies highlight the subjective costs and benefits of self-help group participation. Benefits of program participation include emotional support, shared space for exchanging experiences and information and accessibility due to the online format. Costs include practical barriers as well as emotional distress. Suggestions for increasing the appeal of caregiver interventions for racialized communities include ensuring discrimination-critical facilitation and optimizing the organization of group programs.

**Discussion and conclusion:**

By focusing on subjective costs and benefits, this article seeks to contribute to critical discussions on non-participation research, questioning the practice of labeling certain populations as “hard-to-reach,” while neglecting the factors that researchers and practitioners could change to make program and study participation more appealing.

## 1 Introduction

Approximately 1.84 million people in Germany have dementia (Blotenberg et al., 2023; Deutsche Alzheimer Gesellschaft, 2024). With increasing life expectancy, the number of people over 65 affected by dementia is expected to increase to between 2.3 and 2.7 million (Blotenberg et al., 2023). Data on long-term care needs and dementia prevalence among populations with a history of migration are lacking in Germany. Estimates suggest that around 10% of people requiring long-term care in Germany, a total of 5.7 million people as of 2023 (Statistisches Bundesamt, 2024), have a history of migration (Kohls, 2012; Thum, 2017). With regards to dementia prevalence, estimates suggest that approximately 5.2% of individuals with a history of migration over the age of 65 have dementia and when focusing specifically on first generation migrants from Turkey, a figure of 8,840 affected individuals can be estimated (Monsees et al., 2019).

Individuals with a history of migration in Germany develop long-term care needs approximately 10 years earlier than the general population, in particular due to lower socio-economic status and working lives spent in physically demanding occupations (Bundesministerium für Gesundheit, 2011; Thum, 2017). These risk factors are particularly pertinent to first generation migrants of Turkish descent, who were hired in large numbers as so-called “guest workers” to work in physically strenuous occupations while facing additional stressors in the form of racism and discrimination without systemic supports such as access to language classes (Pfündel et al., 2021).

This context affects family caregiving among individuals of Turkish descent in Germany. Long-term care needs are primarily met by family members in this population group (Kohls, 2012; Krobisch et al., 2014). This is due to a variety of factors, including, but not limited to institutional barriers and negative experiences with public services (Tezcan-Güntekin et al., 2015; Aslan and Tezcan-Güntekin, 2022). Additionally, everyday racism compounds the stress of caregiving, with racialized caregivers frequently experiencing silencing and dismissal by healthcare professionals (Hengelaar et al., 2024). A qualitative study focusing on racialized healthcare users in Germany examined racist encounters with professionals, identifying dynamics in which patients are framed as “others” and have their concerns rendered inaudible by professionals (Gangarova et al., 2024). Additionally, family caregivers of individuals with dementia of Turkish descent report intersecting ableism and racism, for example when professionals reach for racist stereotypes and insults in response to the challenging symptoms of dementia and caregivers’ struggles (Tezcan-Güntekin, 2018). The stress of caregiving may also be exacerbated by sexism. On the one hand, cultural expectations around caregiving, including gendered expectations, can emerge within families (Sagbakken et al., 2018; Annac et al., 2025). On the other hand, racialized sexism appears in anti-muslim discourses framing women of Turkish descent as helpless victims to controlling male relatives, thus denying agency and eliminating nuance in their experiences (Singer, 2012; Coors and Neitzke, 2018; Tezcan-Güntekin, 2018). In this context, it is essential to avoid othering and cultural racism while maintaining a sensitivity to the heterogeneity of people grouped together under the heading of an ethnic group.

Caring for family members with dementia is frequently accompanied by caregiver burden, which can be defined as “the strain or load borne by a person who cares for a chronically ill, disabled, or elderly family member” (Liu et al., 2020, p. 439), with antecedents that depend on caregiver and care recipient characteristics as well as consequences for the well-being of the care dyad. Caregiver burden can be assessed with reference to physical, psychological, social and economic strains associated with the care arrangement. These strains can manifest in a variety of ways, for instance sleep disturbances, mental health problems such as depression and anxiety, social isolation or financial stress (Liu et al., 2020; Lee et al., 2022; Deli et al., 2025). Caregiver burden is unequally distributed, for instance, female family caregivers of individuals with dementia experience higher caregiver burden than male caregivers, which is partly associated with a greater difficulty reconciling caregiving with other daily tasks (Duangjina et al., 2025; De Graaff et al., 2025). It is conceivable that this may be linked to women’s higher overall caregiving responsibilities and the emotional labor expected in various areas of life (Bak McKenna, 2021). Furthermore, a systematic review and thematic analysis of qualitative studies on the caregiving experiences of migrants in Western countries identified barriers to accessing formal care and medical services, including racism and discrimination (Stenberg and Hjelm, 2023). Socioeconomic concerns, as well as the demands of navigating between norms and expectations of migrant communities and wider society, were found to contribute to exacerbated caregiver stress (Stenberg and Hjelm, 2023). However, focusing exclusively on populations with histories of migration risks obscuring the impact of racism on racialized individuals without recent histories of migration. Studies examining the impact of racism on caregiver burden are scarce, though findings from the USA indicate effects of racist discrimination on stress and depression among African American caregivers for individuals with dementia (Cothran et al., 2022). Additionally, low socioeconomic status is associated with increased caregiver burden, including depression and anxiety (Deli et al., 2025). Inequalities thus shape the experience of caring for family members with dementia and influence the level and type of burdens caregivers live with. It is therefore necessary to develop interventions that take specific lived experiences and barriers to access into account to reach out to caregivers who are more likely to be excluded from mainstream care, support and medical services.

Psychoeducational and cognitive-behavioral individual and group interventions are most frequently studied in relation to family caregivers for individuals with dementia with evidence for effective reductions in caregiver burden and depression (Encinas-Monge et al., 2024). Similarly, interventions delivered via technology with a psychosocial focus have been found to be effective in reducing caregiver burden, depression and to a smaller extent, anxiety, while slightly increasing self-efficacy (Cheng et al., 2024). In particular, internet and telephone-based interventions delivered in groups have been found to be effective in reducing caregiver burden and depression (Zhu et al., 2024). However, the utilization of support programs has been found to be low among family caregivers in Germany (Lüdecke et al., 2012).

In the German context, self-help groups are a relatively well-established form of psycho-social support, both for individuals directly affected by chronic illnesses as well as for those caring for them, with approximately 100,000 groups on offer nationwide (Kofahl et al., 2019). A study based on a German sample of chronically ill people and caregivers using self-help groups found that the majority of respondents benefited emotionally and in terms of knowledge acquisition, for instance by feeling less alone and learning from the experiences of others (Kofahl et al., 2019). Not participating in self-help groups was associated with greater social participation, thus potentially lowering the need for further social support. Additionally, education and economic privilege did not differ significantly between participants and non-participants, implying that non-participation is not necessarily a matter of limited information or health-literacy. Furthermore, non-participants cited, among other factors, a lack of need, a lack of appropriate groups in the area and the perception of participation as an added burden as reasons for not taking part (Kofahl et al., 2019).

The availability of, desire for and impact of self-help programs specifically for family caregivers of Turkish descent in Germany has been tentatively examined. A scoping review identified a variety of programs, including information outreach, counseling, supervision of individuals with dementia and self-help groups targeted specifically at people of Turkish descent, however these programs were isolated phenomena rather than part of a wider strategy to include the target group across established services (Aksakal et al., 2020). A qualitative study based on 10 interviews with family caregivers for individuals of Turkish descent living with dementia investigated long-term care arrangements, social support and attitudes toward self-help groups (Tezcan-Güntekin et al., 2019). Respondents expressed a desire for more support from within their social networks as well as from professionals such as doctors or insurance representatives. Attitudes toward self-help groups were ambivalent. While some participants were interested in self-help groups, primarily as a means of knowledge exchange and practical support between caregivers, many expressed skepticism regarding the emotional benefits and the practical costs of participating. Additionally, several participants expressed a desire for remote participation (for instance by telephone), since they would not be able to arrange sufficient time away from caring responsibilities to take part in an in-person program. Concerns were also expressed about regular programs being exclusively tailored to Germans without a history of migration and about not finding enough participants of Turkish descent in smaller regions for a targeted program (Tezcan-Güntekin et al., 2019). An in-person self-help group offered specifically to family-dementia caregivers was conceptualized and piloted following the study outlined above. Participants were interviewed and highlighted emotional benefits, for instance feeling understood by people who have similar life experiences. Learning from the experiences of others was valued highly, as was the opportunity to exchange factual information (Tezcan-Güntekin et al., 2019).

In this context, the Diversity-On project sought to offer online self-help groups to reduce barriers to participation and connect participants from across Germany. The self-help groups were piloted and evaluated, and a non-participation study was conducted. The aims of these two studies were to identify subjective benefits of self-help group participation, find out how to improve the program and gain insight into the subjective costs of participation, while learning how to create more appealing interventions with a more effective reach.

## 2 Materials and methods

### 2.1 Theoretical approach

Reaching individuals who are marginalized, for instance as part of racialized communities, for participation in public health research or health promotion interventions can be challenging. However, individuals tend to have reasons for non-participation that are rational within their lived experience and frequently face barriers to inclusion across a variety of institutional contexts (Escobar et al., 2017; Dingoyan et al., 2022). The term “hard-to-reach” prioritizes the viewpoints of researchers and thus locates the problem with potential participants, rather than inviting self-reflection and an analysis of the ways in which research or intervention projects reproduce structural exclusion. As^9001,47^, p. 2) state: “By disregarding the structural inequalities in society that lead to marginalization, we risk attributing fault to those already marginalized. Denoting nonparticipants with the label ‘hard to reach’ creates a double deficit: denoting them behaviorally and attitudinally deficient, when in fact they face structural social inequalities.” Critical perspectives thus put the onus for change on researchers and professionals rather than potential participants (Escobar et al., 2017; Wöhrer et al., 2020; Pardhan et al., 2025).

In this article we propose a framing that centers intentional choice and agency on the part of potential participants. While choice and agency are often framed in a rational choice paradigm in social and behavioral sciences, critical feminist discourses have called into question the image of the (implicitly male) autonomous, individualist agent purported to be maximizing utility outside of any social context (Herfeld, 2022). Additionally, rational choice paradigms frequently posit forms of economic rationality required within contemporary capitalist societies as essential attributes of human cognition and behavior (Herfeld, 2022). While this article makes use of the concept of a subjective cost–benefit calculation with regards to self-help group participation, this operation is understood to be contingent on the discursive and material conditions caregivers find themselves in. Following a materialist framework that highlights the crucial role of social reproduction (especially care work) in maintaining the economic status quo and intersecting power systems (see Bak McKenna, 2021), this article posits that family caregivers for individuals of Turkish descent living with dementia are restricted and constrained by the material conditions of caring, meaning the resources available to them and the demands on their time and labor capacity. Calculations of cost and benefit are thus not understood as features of innate self-interest or a human predisposition toward maximizing utility, but rather as a response to doing care work under conditions of scarcity (of resources, time and capacity) and at a time when individuals are increasingly expected to submit to market logics and see themselves as autonomous managers of risk and benefit (Cabanas, 2016).

Caregiving in a society in which stressors and resources are distributed unequally along lines of class, race, gender, and able-bodiedness creates conditions in which time and energy for health-promoting activities are scarce, thus necessitating a concerted effort on the part of providers to reduce barriers to access. However, it is also important to acknowledge that family caregivers from racialized communities experience intersectional discrimination in the form of epistemic injustice in healthcare, meaning their capacity to know what is best for them and their family members is routinely called into question (Hengelaar et al., 2024). In this context, it is essential to honor the right to refuse participation in support programs. Overstating vulnerability and exclusion (Kluczyńska et al., 2024) risks denying the agency expressed when individuals intentionally refuse participation. Thus, we take the position in this article that despite challenging material conditions, the target group are experts in their own living situation. It is assumed that they leverage agency and intentionality with regards to participation and non-participation in the online self-help program and that this is reflected in a conscious engagement with the subjective costs and benefits associated with taking part.

#### 2.1.1 Reflexivity in the research process

Reflexivity can be defined as a continuous engagement on the part of researchers with their own social roles and positions as well as with potentially asymmetrical power relations and their effects on data collection, data analysis and the interpretation of results (Pillow, 2003; Berger, 2015). The project Diversity-On was situated in the context of public health research and healthcare provision, in which racialized communities are frequently cast in paternalistic, deficit-focused narratives (Kluczyńska et al., 2024). It was thus important to keep a critical distance from stereotyping and cultural essentialism, while drawing on critical resources in dealing with discriminatory discourses and structures. An approach drawing on the principles of participatory action research was favored with the intention of establishing shared authority (see London et al., 2020) with stakeholders directly involved in providing services for the target group of the evaluation and non-response studies. Close collaboration with cooperation partners providing services for racialized family caregivers helped to attenuate the academic perspectives of the researchers and tailor the research design to the interests of the target group and supporting professionals. Thus, participatory and person-centered principles were taken into account during recruitment and data collection. Additionally, data-analysis was guided by theoretical approaches (outlined above) seeking to shed light on and interrupt hegemonic discourses on migration, care and vulnerability.

### 2.2 Study design

The data presented in this article were drawn from two qualitative studies embedded within a larger research project^1^, whose overall aim was the development, pilot-testing, and evaluation of an online self-help group program for family caregivers for individuals of Turkish descent living with dementia. The results of the qualitative evaluation of the program will be presented alongside the findings from a study with potential participants exploring reasons for non-participation.

The online self-help groups under evaluation began in December 2024 and will continue to be offered beyond the end of the research project in December 2025. Three different groups take place monthly via the videoconferencing platform Zoom (Zoom Communications Inc, 2025). Each group is led by volunteer facilitators of Turkish descent, who have themselves been family dementia caregivers, and accompanied by a professional social worker. Two of the groups communicate primarily in German with the option to speak Turkish whenever desired. The other group communicates primarily in Turkish. In total, 21 participants have attended the groups, however participation varied month-to-month due to the significant commitments faced by the participants and unforeseen events such as severe illness or even death of the family member with dementia. Each group session was begun by the reading of a story representing specific aspects of dementia caregiving among family caregivers of Turkish descent that had been generated in a previous project based on secondary data analysis (Annac et al., 2025).

The qualitative process evaluation was conducted in March 2025, while the final evaluation was carried out in September and October 2025. The research questions guiding the evaluations were:

1 How did participants experience taking part in the self-help groups?
2 How did participants experience the digital communication aspect of the groups?
3 How have perceptions of the groups changed since the process evaluation?
4 Which factors facilitated continued participation and which factors led to drop-out?

Due to initial difficulties in acquiring participants for the self-help groups, the project design was adapted to radically reduce the sample size and investigate potential reasons for non-participation. While originally, the project aimed to recruit 150 participants, the new design set out to pilot-test the group program with 21 individuals. After receiving ethical approval, 78 organizations were approached, of which 13 agreed to help with recruitment. This involved outreach in publications such as newsletters, posting in chat groups and social media, directly approaching members who may be interested in the intervention, or introducing the researchers to other professionals with contacts in relevant communities. By the halfway point of the project, only very few participants had been recruited for the intervention. While many people who were approached for participation showed an interest in the project, they were frequently reluctant to commit to participate, for instance due to time constraints from reconciling caregiving with other activities of daily living. Additionally, it was difficult for cooperating organizations to sustain their recruitment efforts over time. While the researchers in charge of the project had the time and resources to recruit, they were dependent on the networks of the cooperating organizations, who were lacking the time and resources to engage more fully with the project. The tensions inherent in relying on community experts for networking and recruitment as well as the insights gained through working with cooperation partners in the project Diversity-On are reflected on in a publication laying out guidelines for better practice in research with so-called “hard-to-reach” populations (Bilgic et al., 2025).

Alongside reducing the number of required participants, the non-participation study was thus added to the design in order to investigate barriers to participant acquisition and was conducted between June and September 2025. The questions guiding study were:

5 Which factors led potential participants to decide against joining an online self-help group?
6 Which changes to the program would be needed to make participation more appealing?

### 2.3 Sampling and participants

Evaluation interviews were conducted at two different timepoints, each with the same eight participants. To prevent potential overburdening, self-help group participants facing severe current life stressors were excluded from the sample, for instance with severe current life stressors were excluded from the sample, for instance those whose relatives were in life-threatening conditions or those who had chronic illnesses themselves. The remaining group participants were invited to take part in evaluation interviews and eight of them agreed. All eight individuals subsequently participated in the final evaluation.

The age range of the evaluation participants was between 40 and 70 years. All participants were adult children caring for a mother with dementia. Of the participants caring for a mother with dementia, three participants also had fathers with long-term care needs (not dementia related) that added to their caregiving responsibilities (see Table 1). Seven of the participants were women, one was a man (see Table 2).

**Table 1:** Caregiver relationships in the evaluation study.

Caring for mother with dementia	Caring for mother with dementia and father with other long-term care needs
5	3

**Table 2:** Gender of participants in the evaluation study.

Male	Female
1	7

The non-participation interviews were conducted with caregivers who had previously been contacted for participation in the self-help groups and who had declined to participate as well as with individuals who had dropped out. In total, eight interviews were conducted.

The age range of the individuals who took part in the non-participation study was between 35 and 70 years. Seven of eight participants were adult children caring for a parent with dementia – of these, six of were caring for their mothers and one was caring for their father (see Table 3). Seven partipants were female, one participant was male (see Table 4).

**Table 3:** Caregiver relationships of participants in the non-participation study.

Caring for mother with dementia	Caring for father with dementia	Caring for a spouse with dementia
6	1	1

**Table 4:** Gender of participants in the non-participation study.

Male	Female
1	7

### 2.4 Ethical data collection and handling

Prior to data collection for the evaluation and the non-participation study, ethical approval was obtained from the Ethics Committee of Witten/Herdecke University (Application No. S-50/2023). Throughout the data collection process, all participants were informed in writing about the study and the voluntary nature of participation. Informed consent to participate in the study was obtained in writing. All data were handled in accordance with German data protection regulations.

### 2.5 Data collection

Both the process and final evaluations were based on short (10–20 min long) semi-structured interviews. The interviews were conducted in German or Turkish depending on the preferences of the participants. Excerpts were translated into English for this publication by the researchers. All interviews were audio recorded and transcribed verbatim with the aid of noScribe transcription software. NoScribe is a free, open-source program that does not process user data and complies with German data protection legislation (Dröge, 2024).

The interviews were conducted via telephone, since the self-help group participants reside in several different Federal States across Germany. Key sections of the topic guide for the process evaluation were:

1. Positive and negative aspects of the self-help groups
2. Emotional effects of the self-help groups
3. Usability: group organization and digital communication

The topic guide for the second wave of interviews constituting the final evaluation was focused on two main themes: changes in perception throughout the course of the pilot period and reasons for continuities and discontinuities in participation. The researchers were aided in the development of the second topic guide by project partners and supporters in the form of experts from organizations specializing in support for caregivers from racialized and migrant communities. Extensive communication with the experts regarding the barriers faced by the project’s target group led to the formulation of questions specifically relating to difficulties in accessing the self-help groups and about the degree of flexibility needed for participants to be able to integrate group participation into their lives.

Data collection for the non-participation study was also carried out by means of 10–20 min long telephone interviews. Five of the interviews were audio recorded and transcribed using noScribe software (Dröge, 2024). The remaining three interviews were conducted with individuals who agreed to be interviewed, but did not wish to be audio-recorded, so the interviews were recorded in writing by the interviewers. The interview topic guide covered two main topics: Reasons for non-participation and potential incentives for participation.

Since family caregivers are exposed to significant stressors and are frequently burdened by their role, the researchers took precautions in designing and conducting interviews to minimize the amount of time and organization required on the part of the respondents. The topic guides were designed to be short and telephone interviews were planned to minimize the time and effort needed to participate. Prior to data collection, the interviewers agreed on a person-centered approach based on a holistic, reflexive and self-aware approach to responders (Sandvik and McCormack, 2018). Safety was addressed in the form of a plan determining how to respond and who to contact in case participants expressed extreme distress. As part of a critically reflexive approach, the researchers attempted to continuously reflect their roles and potential effects of power differentials on responses by participants. The safeguarding of respondents was prioritized, centering their well-being and letting them set their own agendas, even if this meant veering off the topic-guide.

### 2.6 Data analysis

The evaluation and non-response interviews were both analyzed by means of structuring qualitative content analysis (Kuckartz and Rädiker, 2022). For the evaluation study, deductive categories were arrived at based on the interview topic guides. For the non-participation study, notes from extensive exchanges with experts regarding their experiences around non-participation in health promotion and support programs for family caregivers formed the basis for deductive categories. Inductive sub-categories for both datasets were generated as sections of text were assigned to deductive categories. The sections of text were labeled with sub-category suggestions, which were compared, contrasted, clustered and hierarchized to arrive at final category systems for each dataset. Supplementary Table 1 presents an overview of the category system associated with the evaluation study, including exemplary quotes.

Supplementary Table 2 presents the category system associated with the non-participation study, including exemplary quotes. The experts from organizations supporting family caregivers helped to determine the most relevant aspects of non-participation. They encouraged reflection on the part of the researchers, suggesting an approach that does not frame potential participants as hard-to-reach, but rather highlights barriers and subjective costs associated with programs that may not meet their needs.

## 3 Results

Not all categories and sub-categories will be exhaustively presented in the results section. The bold-formatted categories and sub-categories (see Supplementary Tables 1, 2) will be addressed in this article. A composite category has been created based on “Critique and suggestions for improvement” and “conditions of participation” to synthesize findings from both studies concerning ways to increase the appeal of online self-help group participation. The composite category was labeled “Increasing the appeal of online self-help group participation” with the sub-categories “organization” and “organizational conditions” being combined into a single subcategory. “Discrimination-critical spaces” was retained as a sub-category.

### 3.1 Benefits of online self-help group participation

Evaluation participants described their experiences in the self-help groups overwhelmingly in positive terms. In particular, the emotional benefits, the function of the groups as a space for sharing experience and expertise as well as the benefits of the online format were highlighted. Several participants mentioned that their initial worries about participation, for instance about not receiving enough information or not being able to trust people, were not confirmed. As IPE4.1^2^ stated: “I think others might feel like I did at the beginning – they might not know if they can trust people. Now I feel safe and I can really say that I am able to share very personal things” (IPE4.1, 174–180). The fact that the benefits of participation were able to outweigh initial skepticism is a strong endorsement of the efforts made to acquire participants and implement the intervention. However, these findings relate to individuals who stayed in the groups and they should not be considered in isolation from the concerns expressed by individuals who left or declined to participate (see costs of participation).

#### 3.1.1 Emotional benefits

All participants reported beneficial effects of program-participation on their emotional wellbeing, including a sense of relief after meetings, feeling more in touch with emotions and feeling less lonely. Several participants emphasized positive changes in mood after each meeting, highlighting that they felt less burdened after spending time with others sharing similar caregiving experiences. One participant stated she was able to feel and process difficult emotions that she suppressed in everyday life when other self-help group participants shared their feelings:

“I often notice that when others talk about themselves, when I realize I am also affected by the same things, right? With the topics discussed by the others, I realize that I also feel sad or start to get emotional, my feelings come up, the ones I normally suppress, I think I start to experience them. I don’t cry or anything like that, but I notice that I get more emotional and start experiencing these feelings and I think that’s a good thing.” (IPE2.1, 34–40).”

Several participants stated that they felt empowered after meetings and better able to cope with difficult care arrangements. Two participants referred to the self-help group as therapeutic or healing. Additionally, multiple participants mentioned benefitting from a sense of belonging during meetings. The self-help groups were regarded as a way of experiencing a sense of community and a means of combatting loneliness. For instance, IPE5 stated that she feels less alone due to her participation in the group and feels more balanced after meetings:

“I would almost say [the self-help group] is healing, because the emotions, the feelings, the thoughts one has in everyday life, one often feels alone with these thoughts, even though one has a family, friends, colleagues. Yes, but the others are in a different place with their feelings and their thoughts and that’s where our meetings are really good. I always feel very calm and internally organized after the meetings.” (IPE5.1, 6–12).

Overall, the self-help group participants derived significant emotional benefits from participating in the program.

#### 3.1.2 Creating and holding space for shared experience and expertise

Most participants expressed an appreciation for the self-help groups as reliably available spaces for sharing their experiences. The volunteer facilitators were praised for their abilities in creating an open and validating environment as well as for providing a reliable port-of-call for questions and concerns. The group was regarded as a space for exchanging information and experiences as well as learning from each other in coping with difficult care arrangements. Many participants explicitly valued the expertise, both professional and experience-based, of their fellow members and of the facilitators. Professional expertise was contributed by some of the participants who had qualifications in medicine, long-term care or social work, while also being family caregivers. One participant voiced appreciation for the group as a platform for learning and information exchange:

“And in this phase [of dementia] it was super helpful just to explain what’s going on, what we’ve already done, what we don’t know yet, what comes next. And I got some very helpful advice from the group that I then implemented. […] So that was really- to get feedback from other caregivers was great because they understood and the experts in the group gave me great advice that helped a lot.” (IPE1.1, 43–52).

Another aspect of the space provided by the program that was valued by participants was the focus on caregivers of Turkish descent. This sentiment is expressed by IPE1.1:

“I really appreciate that the program is specifically for people who speak Turkish. I’ll put it like this, you’re in a group with a shared cultural background. And you just understand each other at times without needing to explain so much. And that makes everything a bit easier, all in all.” (IPE1.1, 26–31).

The self-help groups thus gave participants space to exchange information and experiences in a relatively safe environment, without having to field potentially inappropriate or intrusive questions regarding culture or ethnicity.

#### 3.1.3 Benefits of the online format

During the final evaluation, participants were asked more explicitly about advantages and disadvantages of the online format of the self-help program. All participants highlighted the positive aspects of the online format, in particular the opportunity to meet with people from all over Germany and the ease of joining meetings regardless of location. For instance, IPE2.2 stated:

“A significant advantage of the digital format is that people who would otherwise not be able to reach each other are able to get together. I think for me that was a really important aspect. That way it’s possible for someone who lives in Hamburg, for example, to join and we all come together.” (IPE3.2, 76–79).

Additionally, the participants emphasized how the digital format made self-help group participation more accessible for them. They highlighted how important it was for them to be able to participate from any digital device, including their phones, from any location that was convenient. For instance, one participant mentioned that an in-person group would have interfered with child-care, while the online format allowed her to participate from her living room while her daughter played elsewhere in the house.

Furthermore, one participant mentioned that she felt it was easier to open up to people in the group, who had initially been strangers, online rather than in person:

“Maybe there’s less of an emotional threshold when opening up to strangers online as opposed to going somewhere in person, I do think it’s easier for me. Talking to strangers on the screen was, in fact, easier for me.” (IPE1.2, 139–140).

While two participants mentioned that technical barriers may exist for older family-members, who may also benefit from a support program, they themselves all felt very comfortable using video-conferencing technology. Since the evaluation interviews were conducted with individuals who regularly participated in the online program, there is a bias in relation to technological affinity in the sample. While limited digital literacy can constitute a barrier for participation in online-programs, it is worth noting that none of the individuals interviewed in the non-participation study cited the digital nature of the program as a significant reason for not joining the self-help groups. It is thus important not to generalize based on ageist or racist assumptions that digital literacy must be low in certain population groups.

### 3.2 Costs of online self-help group participation

#### 3.2.1 Practical costs

All participants mentioned a lack of time as a key factor preventing them from participating in the self-help group. Caregiving activities alone were described as taking up a large amount of time, including activities directly involving the family member with dementia such as supervising meals or changing sheets in case of incontinence as well as organizational activities to ensure medical care and seamless supervision. Many of the respondents had additional responsibilities, especially employment and under-age children, in many cases both. Completing all of the tasks associated with these responsibilities was associated with a high mental load, meaning that family caregivers invested considerable cognitive and emotional resources in planning, coordinating and reconciling tasks in different areas of their lives. Many of the participants emphasized that any additional activities, even activities that might be beneficial to emotional well-being, would add to the burden of organizing and completing tasks in their busy day-to-day lives. One participant emphasized that the practical and mental load was particularly high during the advanced phase of dementia their relative was in, thus making it even more difficult to incorporate a new commitment into their schedule.

Several participants emphasized that their familial duties, such as spending time with children, took priority over any potential new plans. Having a demanding job was also cited as a reason to reject self-help group participation, as IPN^3^1 (106–108) explains: “Now I’m also dealing with a challenging employment situation, that’s happening on top of everything. If I was in a phase in my life in which I could be generous with my time, that might have an influence [on the decision to participate].”

The decision to participate in a self-help group is thus embedded in the context of the multiple competing demands that family caregivers face in everyday life. Frequently, it is difficult to step away because they play integral roles in day-to-day caregiving and have tight schedules to reconcile tasks that need to be done in different areas of life. Additionally, time to oneself is scarce for family caregivers and individuals may decide against taking up another structured activity. IP7 summarizes the steps that can be necessary to clear a time-slot for a new appointment or commitment:

“For every new additional appointment, not just self-help group meetings, but any appointment, I have to plan it, discuss with my siblings, decide who will take him to the clinic, who will come over when it’s time for my appointment.” (IPN7, 227–235).

Two participants stated that they did not feel a need to talk to others about dementia caregiving or to share experiences. In relation to the day-to-day responsibilities demanding their attention, they assigned a low priority to this type of social activity. Both participants highlighted their openness to participate in an intervention program in the future, for instance at a different stage of their family member’s illness. However, the stress of making time for another appointment outweighed the desire to talk at the time of the interview.

#### 3.2.2 Emotional costs

Self-help group participation was linked linked to an expected emotional cost by several of the respondents in the non-participation study. For instance, shame was mentioned as a barrier to participation as well as stress caused by the commitment to attend on a regular basis. A perceived discrepancy between one’s own situation and that of others in the group led some individuals to reject (further) participation. One participant was reluctant to rejoin after attending one meeting and discovering that all the other attendees had grown up in Germany and felt comfortable speaking German and occasionally switching to Turkish, while she herself had migrated as an adult from Turkey and would have felt more comfortable in a primarily Turkish-speaking group. Another participant, who had not attended any meetings, similarly mentioned that she feared encountering language barriers in the program. These findings highlight the importance of accounting for heterogeneity within target-groups for health promoting interventions to avoid homogenizing and stereotyping while creating inclusive environments for a variety of participants.

One participant perceived a discrepancy between himself and other participants with regards to the stage of illness his mother was in. IPN1 describes:

“I heard the other participants’ stories in this meeting and that really shook me up emotionally. For me it triggered the following: ‘Oh god, I hope that doesn’t happen to me’. I think it’s a bit inappropriate to listen to the suffering of others and to think ‘thank god I’m doing better than them’.” (IPN1, 93–100).

Non-participation can therefore be a conscious decision undertaken by individuals for a variety of reasons, including to protect themselves from emotional harm.

### 3.3 Increasing the appeal of online self-help group participation

This section synthesizes findings from the evaluation and the non-participation study to identify features desired both by individuals who did and by those who did not participate in the self-help program.

#### 3.3.1 Organization

One of the suggestions for improvement regarding organizational aspects produced a dilemma: respondents in both studies expressed a desire for consistent attendance, but they also highlighted the importance of flexibility due to the extensive demands on their time from caregiving and other day-to-day commitments. Some self-help group participants also voiced a desire for larger groups to compensate for low or inconsistent attendance. This highlights the necessity to continuously recruit participants for programs like the one considered in the present study. In addition to inconsistent attendance due to demanding day-to-day lives, several participants dropped out of the program over the course of the pilot period due to changes in the health status or even the death of their relative with dementia. Facilitators thus need to be sensitive when following up with participants who stop attending family-caregiver programs.

If participant turnover increases, so may concerns over confidentiality. Several participants mentioned initially being wary of sharing personal information with strangers and desired reassurance of confidentiality.

“Because, essentially, you do a sort of ‘strip-tease of the soul’. The problem is, to what extent is it a protected space, is it safe for me. I had to ask again. That’s very important for me, because you talk about very personal, very private things.” (IPE7.2, 14–18).

One of the participants suggested that facilitators and participants sign confidentiality agreements before commencing the program. This may be a way of increasing the appeal of participation, especially if fear of disclosure and shame are experienced as barriers.

#### 3.3.2 Discrimination-critical spaces

Several participants in the non-participation study mentioned that a condition for them participating in a self-help group program would be an explicit effort on the part of organizers to prevent discrimination. Several participants mentioned cultural sensitivity, for instance IPN4 explained: “it would be important to me that the [organizer] is knowledgeable about the culture, not just reading something off a paper, but showing real understanding, maybe also from personal experience” (IPN4, 40–46). Another participant mentioned homogenizing and stereotyping attitudes and expressed that he would expect organizers to see participants as individuals:

“[Ideally, in a self-help group setting] one wouldn’t be labelled immediately. A lot of people think: ‘The Turks are like this or like that’. I just want to be seen as a person, not as a cliché. To be able to speak openly, without the fear of being misunderstood”. (IPN 8, 47–49).

While the respondents express a desire for their cultural backgrounds to be addressed with sensitivity, it is equally important not to reproduce cultural racism and essentialism. Participants should not be confronted with pressure to represent their ethnic group when attending health promoting programs. Another participant mentioned during the interview that she had frequently experienced discrimination and exclusion in the healthcare system while seeking medical care for her mother with dementia. In this context, it is particularly important to create discrimination-critical spaces in self-help groups addressing individuals from marginalized communities.

## 4 Discussion

The present study identified several benefits of online self-help group participation from the perspective of family caregivers of Turkish descent. The interview respondents highlighted benefits to emotional well-being, including feeling understood by other group members and felling less lonely. Group participation was described as emotionally moving and even as healing. These findings correspond to the benefits of self-help group participation identified by Kofahl et al. (2019) and allow for an insight into specific factors that may underpin improvements in caregiver burden or depression identified in relation to in-person and technologically mediated programs for dementia caregivers (Cheng et al., 2024; Encinas-Monge et al., 2024; Zhu et al., 2024).

The evaluation respondents also emphasized the benefits of exchanging experiential and expert knowledge and thus learning more about how to manage their family members’ condition. They were able to receive information that was useful and relevant to them in their particular caregiving experience and felt that they benefitted from the expertise of group members and facilitators. Knowledge exchange is an established benefit of interventions and self-help groups for family caregivers, including those designed specifically for individuals of Turkish descent (Kofahl et al., 2019; Tezcan-Güntekin et al., 2019; Encinas-Monge et al., 2024). The results presented here contribute insights into self-help groups as inclusive epistemic environments.

Racialized and otherwise minoritized groups are often labeled as vulnerable in research, which can read as a paternalistic, stereotyping view casting people as victims or limited in their competency (Kluczyńska et al., 2024). Additionally, their capacity to know and make accurate judgments as caregivers is called into question (Hengelaar et al., 2024). In this context, it is important to highlight that the self-help group participants emphasized the importance of sharing experiential and professional knowledge during meetings. While many participants had professional backgrounds, for instance in medical and social fields, that allowed them to contribute highly valued expert knowledge to group meetings, the value of lived experience was emphasized just as emphatically, suggesting an environment allowing for greater epistemic justice than other healthcare contexts (Gangarova et al., 2024; Hengelaar et al., 2024).

The accessibility provided by the online format of the self-help groups was unanimously praised by the evaluation participants. They particularly appreciated the flexibility of being able to participate from any location, for instance by using mobile devices, and were thus able to better integrate the groups into their daily lives. Scheduling conflicts that may emerge for in-person meetings could be avoided, for instance allowing participants to be present in the home for childcare. Since they all made the decision to join the groups, knowing they would be held online, the sample is biased in this regard. Nevertheless, in light of previous research showing that a lack of appropriate regional groups as well as concerns specifically from caregivers of Turkish descent about finding time for participation or being reluctant to use services where they are minoritized (Kofahl et al., 2019; Tezcan-Güntekin et al., 2019), the online-format seems to resolve some of the barriers excluding dementia-caregivers from self-help groups.

The respondents of the non-participation study emphasized practical costs of participation, in line with findings that non-participation in a self-help group context can be related to perceptions of attendance as an additional burden in daily life (Kofahl et al., 2019; Tezcan-Güntekin et al., 2019). Additionally, several participants highlighted emotional reasons for not participating, showing that non-participation can result from a conscious decision to protect oneself from emotional harm. The decision not to participate in the self-help groups was regarded as an expression of agency and subjective rationality, while remaining attentive to evidence of structural exclusion and opportunities for improvement on the part of researchers and intervention providers. A key theme that emerged in the category regarding increasing the appeal of the online self-help program was the expectation that interventions targeting racialized communities should be critically reflexive with regards to discrimination as well as sensitive to heterogeneity within the target group. As in other studies on intervention utilization by racialized individuals, the participants referred to instances of racism experienced in the context of healthcare (Dingoyan et al., 2022; Gangarova et al., 2024; Hengelaar et al., 2024). It is important to keep in mind that target groups defined as vulnerable or hard-to-reach are capable of analysis and reflexivity in relation to the services they use and refuse. Lived experience yields knowledge used to evaluate and decide whether needs will be met or dismissed or whether people and communities will be represented sensitively and accurately or with recourse to stereotypes (see Hengelaar et al., 2024).

Organizational aspects are a key area in which researchers and providers of intervention projects can reduce barriers to participation. Flexible attendance policies are required by family caregivers for individuals with dementia who may frequently miss appointments due to urgent needs on the part of their relatives or who may drop out due to a worsening of the illness or death. Continuous recruitment is therefore necessary to compensate for the turnover. Working with the contradiction between the desire for a continuous and reliable group-program and the need for flexibility in attendance is a challenge for organizers. It is advisable in this context to take heed of the “seven Cs” of participatory action research: 1. cyclical, 2. collaborative, 3. context-specific, 4. combining theory and practice, 5. critically reflective, 6. change-focused and 7. conversation-driven (Feekery, 2024). Diversity-On was conceptualized with recourse to participatory principles, involving frequent, high-intensity cooperation with cooperation partners providing services to the target group. As such, the guiding principles above were taken into account and simultaneously, the researchers learned to better interpret and apply them throughout the project. Especially the cyclical, collaborative and critically reflective aspects were essential for responding to challenges such as the dilemma described above. The situation would feed back into decision-making on the part of the researchers and cooperation partners, who would develop solutions collaboratively and critically reflect on how to move forward in a way that would optimally support the change-focused orientation of the project with the aim of easing the burden experienced by the participating caregivers.

### 4.1 Limitations and suggestions for future research

The difficulties in reaching interview participants mirrored difficulties reaching potential self-help group participants. The researchers attempted to minimize the time and effort – and correspondingly the cost – associated with participating in interviews. However, there may not have been enough of a benefit to potential participants to legitimize agreeing to an interview. A more stringently participatory approach may have helped, for instance self-help group participants, who formed the sampling pool for the evaluation study, could have been involved in choosing the data collection method for the evaluation. Perhaps they would have preferred a focus-group, conducted immediately after a group meeting, or a written evaluation format to eliminate the need for more appointments. Future research efforts may benefit from leaving certain research design considerations open to ensure more opportunities for potential participants to share decision-making. Reaching participants for the non-participation study was even more difficult and was only possible at all by drawing from the pool of individuals who had dropped out of the groups and by working closely with cooperation partners and their networks. The latter strategy requires significant time and relationship building on the part of researchers and should be included in research designs at the earliest possible stage to allow for trust-building and reciprocal working relationships.

The samples of both studies were small and heavily biased toward women. Potential gender-based differences and intersections with migration experiences could therefore not be explored in depth. Additionally, adult children caring for parents with dementia were overrepresented, particularly adult children caring for their mothers. Women are overrepresented among family caregivers and are more likely to use support interventions than men (Lüdecke et al., 2012). Additionally, spousal caregivers have been found to be more reluctant to use support services than adult children and are likely to experience higher caregiver burden (Friedemann and Buckwalter, 2014). Potentially, the bias in the samples presented here reflect these tendencies. However, more intersectional research is needed to identify the potentially different needs and expectations different caregiver groups may have with regards to interventions in order to tailor programs and recruitment strategies more effectively.

Furthermore, it is important to note that the participants of the evaluation study may have felt a particular pressure to offer socially desirable answers regarding the benefits of the program, since they had been involved in the groups for some time and may not have wanted to offend the group coordinator and members of the research team, whom they had become acquainted with.

Another aspect to consider is the impossibility of guaranteeing completely discrimination-free spaces. While the social worker coordinating meetings and the volunteer facilitators were well-versed in discrimination-critical, reflexive practices, the same degree of reflexivity could not be expected from every group participant. The Diversity-On self-help groups were experienced as positive, safe, and respectful environments where participants felt comfortable sharing details of their private lives and their feelings. This atmosphere can be protected and nurtured by facilitators, but the risk of offence or discrimination cannot be eliminated entirely. This should be considered when planning similar projects with safeguarding precautions in place.

Finally, regarding the focus of the studies on individuals of Turkish descent, it is important to emphasize that the findings presented here cannot easily be generalized to other racialized or migrant groups without risking homogenization.

## 5 Conclusion

The results presented in this article clearly show that online self-help groups have the potential to provide a variety of emotional, social and practical benefits to family caregivers for individuals with dementia from racialized communities. Program participants valued the exchange of information, emotional relief and sense of community associated with the self-help group meetings. At the same time, both studies exposed the challenges associated with regular participation in a health-promoting intervention program. Family caregivers often navigate multiple different roles, reconciling demands on their time and energy from the unpredictable nature of caregiving duties with other care responsibilities and paid employment. The highly private nature of concerns around caregiving leading to difficulties trusting the setting can also limit willingness to participate. In this context, a monthly online-meeting can be perceived as an additional burden and demand – both emotionally and practically. Finally, this article highlights that non-participation in a health-promotion intervention should not be framed as a deficit on the part of the target group, but rather as the expression of a subjective reckoning with the structural barriers of taking part. Furthermore, a critically reflexive approach involves researchers and professionals taking responsibility for creating accessible and appealing programs and research projects conceptualized with participatory and discrimination-critical principles in mind. Finally, this article contributes a critical approach to non-participation grounded in recognizing the agency and epistemic empowerment of individuals from racialized communities in determining what is best for them.

## Data Availability

The datasets presented in this article are not readily available because the sharing of the raw qualitative interview data was not specified in applications for ethical approval or data protection statements. The raw data will therefore not be made publicly available. Requests to access the datasets should be directed to rona.bird@proton.me.

## Footnotes

1 Diversity-On: Prävention und Gesundheitsförderung für pflegende Angehörige. Entwicklung und Evaluation eines diversitätssensiblen Online-Selbsthilfeangebots zur Stärkung des Selbstmanagements am Beispiel von Menschen mit türkischem Migrationshintergrund.

2 IPE, Interview Participant Evaluation. The evaluation participants are coded IPE1-IPE8. The process evaluation interviews are coded IPE#0.1, while final evaluation interviews are coded IPE#0.2.

3 IPN, Interview Participant Non-participation study. The participants are coded IPN1-IPN8.
