# Supplementary material for: Subjective costs and benefits of online self-help group participation: findings from qualitative research with family caregivers for individuals of Turkish descent living with dementia in Germany
Source: Front Dement. 2026 May 21;5:1763257. doi: 10.3389/frdem.2026.1763257 (PMC13233225; doi:10.3389/frdem.2026.1763257)
Supplement: Supplementary file 1 [file Table_1.docx]

**Supplementary table 1: Category system for the evaluation study**

| **Deductive categories** | **Inductive sub-categories** | **Exemplary quotes** |
| --- | --- | --- |
| **Benefits of online self-help group participation** | **Emotional benefits** | “Like I said, I do feel relieved, like I said, I can talk about topics that- I can’t talk about everything with everyone. Not everyone would understand me […], it feels good to be able to just talk through some things. It reduces the load when one can just complain a bit.” (IPE7.1, 96-100) |
|  | **Creating and holding space for shared experience and expertise** | “It feels good to interact and get valuable tips from other family caregivers who might also be trying out new things in their everyday lives, or one can give advice and talk about one’s own situation without getting know-it-all responses” (IPE6.2,19-25) |
|  | **Benefits of the online format** | “An advantage [of the online format] is that I can participate from anywhere, even from my phone. I don’t see any disadvantages, honestly. It is also an advantage that we can interact with people, who don’t live close by” (IPE8.2, 58-67) |
| **Critique and suggestions for improvement** | **Organization** | “I think that these groups need to be advertised more. I have a friend whose mother has dementia and she has never heard of this kind of organization and doesn’t know how to participate in these kinds of groups […] I think you should advertise the groups more” (IPE6.1, 48-53) |
|  | Content | [In response to a question about suggestions for improvement] “What is most important to me is bureaucracy. That is an area I am uncertain about, to be honest. Where can I get financial support? What do I need to apply for? What am I doing right? What am I doing wrong? These things are important to me.” (IPE7.1, 50-56) |
| Usability aspects | Digital communication | “[The organizer] does an excellent job. We decided to start a Whatsapp chat for our group, which exists in addition to the information we receive by email. Due to the Whatsapp group, everyone stays informed, even if they didn’t get around to checking emails that week. [We receive] another reminde via Whatsapp: tomorrow or the day after tomorrow the zoom call will take place. And that is really great.” (IPE3.2, 57-62) |
|  | General organizational aspects | “And since I knew the group appointments for the rest of the year or for the next six months, I wrote to my boss very early on to tell her on which days I would need to start work later. And then it worked out.” (IPE5.2 33-36) |
