# Supplementary material for: Subjective costs and benefits of online self-help group participation: findings from qualitative research with family caregivers for individuals of Turkish descent living with dementia in Germany
Source: Front Dement. 2026 May 21;5:1763257. doi: 10.3389/frdem.2026.1763257 (PMC13233225; doi:10.3389/frdem.2026.1763257)
Supplement: Supplementary file 2 [file Table_2.docx]

**Supplementary table 2: Category system for the non-participation study**

| **Deductive categories** | **Inductive sub-categories** | **Exemplary quotes** |
| --- | --- | --- |
| Stressors and the lived experience of caregiving | Reconciling multiple demands | “And since I work full time, it was mainly the time that prevented me from joining […] Then it [the group] was on Wednesdays and that is my only free afternoon. And then I always had physiotherapy, because I scheduled all my physio appointments for Wednesdays because I didn’t think of it [the group] at first, you know?” (IPN3, 15-21) |
|  | Mental load | “And then one has to, sometimes it was a difference like day and night, take care of a child as well and make sure that’s going well. Also to negotiate with all of the other parties, with family members, professionals, everyone involved. And then of course one always worries that something will go wrong, that he will injure himself, get lost” (IPN7, 154-159) |
|  | Giving it all as a caregiver | IPN2 struggled with the group appointment in the morning before lunch. She particularly wants to participate in the care of her mothers in the morning when she needs physical care and lunch is being prepared. She says “the others [family members, care professionals] do not have as much patience as me, even my brother in law, who is a doctor. I want to treat her like a little child, I want to make the effort”. She wants to provide care in a “humane” way, “with heart”. (IPN2, excerpt from notes for an interview that was not recorded – see methods) |
| **Costs of online self-help group participation** | **Practical costs** | “It depends on the person, whether they need it [the group] or not. Some family caregivers are very burdened and might look for interaction or a third opinion. That can be helpful. But for me the problem is about day to day life – one has so much to do that one thinks about that sort of thing [self-help group] last.” (IPN4, 15-19) |
|  | **Emotional costs** | “Shame also plays a role sometimes – many people in my generation do not like to talk about private matters.” (IPN8, 15-16) |
| **Conditions of participation** | **Organizational conditions** | “When I’m acutely in need, I think meetings once or twice a week make sense […]. In one month so much happens and then you might not remember much, so maybe something in between, maybe every 14 days, that’s how I feel.” (IPN1, 37-41) |
|  | **Discrimination-critical spaces** | IPN6 listed the following as conditions of participation: interaction on equal footing, respectful treatment of all participants by organizers, cultural sensitivity and multilingual groups (IPN6, excerpt from notes for an interview that was not recorded – see methods) |
